# A single-center retrospective cohort study of perioperative systemic chemotherapy in diffuse malignant peritoneal mesothelioma

**DOI:** 10.1371/journal.pone.0275187

**Published:** 2022-09-29

**Authors:** Xiao Wang, Sharyn Katz, John Miura, Giorgos Karakousis, Leonid Roshkovan, Suzanne Walker, Sally McNulty, Christine Ciunci, Keith Cengel, Corey J. Langer, Melina E. Marmarelis

**Affiliations:** 1 Division of Hematology & Oncology, Department of Medicine, University of Pennsylvania Health System, Philadelphia, Pennsylvania, United States of America; 2 Department of Radiology, University of Pennsylvania Health System, Philadelphia, Pennsylvania, United States of America; 3 Division of Endocrine & Oncologic Surgery, Department of Surgery, University of Pennsylvania Health System, Philadelphia, Pennsylvania, United States of America; 4 Department of Radiation Oncology, University of Pennsylvania Health System, Philadelphia, Pennsylvania, United States of America; Fondazione IRCCS Istituto Nazionale dei Tumori, ITALY

## Abstract

**Background:**

Diffuse malignant peritoneal mesothelioma (DMPM) is a rare variant of malignant mesothelioma, representing 10–15% of malignant mesothelioma cases. The preferred therapeutic approach is cytoreductive surgery (CRS) accompanied by hyperthermic intraperitoneal chemotherapy (HIPEC); the role of systemic chemotherapy is not well established. While some limited retrospective studies report worse outcomes with neoadjuvant chemotherapy, our institution has favored the use of neoadjuvant chemotherapy for symptom relief and surgical optimization. The aim of our study was to assess the outcomes of patients receiving neoadjuvant chemotherapy, compared to those receiving adjuvant or no perioperative chemotherapy.

**Patients and methods:**

We conducted a single-center retrospective cohort study of treatment-naïve, non-papillary DMPM patients seen at our institution between 1/1/2009 and 9/1/2019. We explored the effect of type of systemic therapy on clinical outcomes and estimated median overall survival (mOS) using Kaplan-Meier curves. Hazard ratios (HR) calculated by Cox proportional hazard model were used to estimate effect of the exposures on overall survival.

**Results:**

47 patients were identified with DMPM (median age at diagnosis 61.2 years, 76.6% epithelioid histology, 74.5% white race, 55.3% known asbestos exposure). CRS was performed in 53.2% of patients (25/47); 76.0% of surgical patients received HIPEC (19/25). The majority received systemic chemotherapy (37/47, 78.7%); among patients receiving both CRS and chemotherapy, neoadjuvant chemotherapy was more common than adjuvant chemotherapy (12 neoadjuvant, 8 adjuvant). Overall mOS was 84.1 months. Among neoadjuvant patients, 10/12 underwent surgery, and 2 were lost to follow-up; the majority (9/10) had clinically stable or improved disease during the pre-operative period. There were numerical more issues with chemotherapy with the adjuvant patients (4/8: 2 switches in platinum agent, 2 patients stopped therapy) than with the neoadjuvant patients (2/10: 1 switch in platinum agent, 1 delay due to peri-procedural symptoms). Neoadjuvant chemotherapy was not associated with worse mOS compared to adjuvant chemotherapy (mOS NR vs 95.1 mo, HR 0.89, 95% CI 0.18–4.5, *p* = 0.89).

**Conclusions:**

When used preferentially, the use of neoadjuvant chemotherapy in DMPM patients was not associated with worse outcomes compared to adjuvant chemotherapy. It was well-tolerated and did not prevent surgical intervention.

## 1 Introduction

Diffuse malignant peritoneal mesothelioma (DMPM) is a rare disease, representing 10–15% of malignant mesothelioma cases. Patients present with both local and systemic symptoms, including abdominal pain and distension, ascites, bowel obstruction, weight loss, anorexia, and fatigue [1–3]. Compared to patients with malignant pleural mesothelioma, DMPM patients represent a younger and predominantly female population [2, 4] and have a more favorable prognosis [5, 6]. While asbestos exposure remains a strong environmental risk factor, there is a weaker relationship (33–50% vs. >80%), with a shorter latency period between exposure and disease onset (20 years vs. 30–40 years) compared to the relationship with asbestos observed in malignant pleural mesothelioma patients [2].

DMPM cases have three primary distinct histologic subtypes: epithelioid, sarcomatoid, and biphasic. Epithelioid is the most common and is associated with improved survival compared to other subtypes [4–7]. A fourth subtype–well-differentiated papillary mesothelioma–is associated with even more favorable outcomes [8], though data are limited. Deletions of *BRCA*-associated protein 1 (BAP1) have been reported in 50–60% of DMPM specimens; *BAP1*^*del*^ mutations are also associated with improved overall survival [9, 10]. Finally, while disease burden remains an important prognostic factor, staging is a challenge. While there are several proposed surgical and radiologic staging systems [7], there remain inconsistencies in staging between different imaging modalities [11], as well as between “true” pathologic tumor involvement compared to intraoperative estimations [12], making these metrics unreliable and unstandardized across clinical practices.

The preferred therapeutic approach in epithelioid histology DMPM is treatment at a referral center with cytoreductive surgery (CRS) with hyperthermic intraperitoneal chemotherapy (HIPEC) [2, 13–15], which extends median overall survival (mOS) from 6–7 months with no treatment to 34–92 months with CRS/HIPEC. The benefits of systemic chemotherapy are unclear, and the timing of therapy around surgery (neoadjuvant vs. adjuvant) remains controversial. An initial landmark study with 401 patients [13] presented data suggesting benefit from systemic chemotherapy in the perioperative setting, prompting some centers to begin using adjuvant or neoadjuvant chemotherapy. This is similar to the treatment of malignant pleural mesothelioma, where both neoadjuvant and adjuvant approaches are used for surgically resectable disease.

Subsequently, several retrospective studies have compared adjuvant and neoadjuvant chemotherapy with mixed conclusions (Table 1). An analysis of DMPM patients from France [Kepenekian et al., 16] reported a drastic difference in mOS between neoadjuvant and adjuvant patients, while a more recent US study [Bijelic et al., 15] showed a more modest impact on survival. Importantly, for both studies, the type of surgery (CRS + HIPEC versus CRS alone) was not standardized. In contrast, two other studies [17, 18] showed no difference in survival between these two groups. Notably, retrospective studies comparing neoadjuvant versus adjuvant chemotherapy likely suffer from a bias favoring adjuvant chemotherapy since patients offered neoadjuvant chemotherapy often have a higher disease and symptom burden and are initially not optimal surgical candidates. There are no prospective randomized studies comparing these two treatment options.

**Table 1:** Summary of existing literature regarding neoadjuvant vs adjuvant chemotherapy in DMPM.

Study	Years	Setting	Outcomes
Kepenekian et al. [16]	1991–2014	France, RENAPE database	neoadjuvant (*n* = 42) mOS 37 months vs. adjuvant (*n* = 16) mOS 82 months
Deraco et al. [17]	1995–2011	Italy, single institution	neoadjuvant (*n* = 60) vs. adjuvant/no chemotherapy (*n* = 56), no difference in mOS, possible difference in mPFS (*p* = 0.08)
Bijelic et al. [15]	2003–2014	United States, National Cancer Registry	neoadjuvant (*n* = 50) mOS 27.9 months vs. adjuvant (*n* = 266) mOS 35.2 months
Naffouje et al. [18]	2004–2014	United States, National Cancer Registry	neoadjuvant (*n* = 55) mOS 52.3 months vs. adjuvant (*n* = 228) mOS 55 months

In our study, we aim to evaluate the outcomes and characteristics of DMPM patients treated with neoadjuvant chemotherapy. At our institution, DMPM patients who present at diagnosis or for consideration of surgery are routinely offer neoadjuvant chemotherapy, regardless of disease burden, to reduce symptoms and improve the ability to perform complete debulking. This has largely been driven by expert opinion and our institutional experience. Given the conflicting outcomes of patients receiving neoadjuvant chemotherapy as described above, we conducted a retrospective cohort study of DMPM patients treated at our institution to assess clinical outcomes, specifically compared to patients treated with adjuvant chemotherapy or no perioperative chemotherapy.

## 2 Method

A retrospective cohort study was performed of DMPM patients treated at the University of Pennsylvania Health System. All DMPM patients treated at our institution between January 1, 2009 and September 1, 2019 were identified using ICD9/10 codes (C45.9, C45.1, C45.7). Patients with well-differentiated papillary DMPM were excluded. Electronic health record abstraction was used to confirm pathologic diagnosis and record demographics (age, sex, smoking history, asbestos exposure, Eastern Cooperative Oncology Group performance status [ECOG PS]), treatment history (surgery including CRS/HIPEC, systemic chemotherapy course), and clinical outcomes (survival, radiographic response). Patients undergoing neoadjuvant and adjuvant chemotherapy were further analyzed to assess comparisons between these two groups, including pre-treatment labs, pre-treatment tumor burden, presence of ascites, and pre-diagnosis Charlson Comorbidity Index (CCI). Response to neoadjuvant chemotherapy was assessed using RECIST 1.1 criteria. Of note, given limitations in staging as described above, our institution did not routinely utilize a staging system such as Peritoneal Cancer Index (PCI) during the 10-year study period; thus, this was not extracted or analyzed in our study.

Statistical analysis was performed using STATA software (version 14.2, StataCorp). Comparisons between groups utilized Student’s t-test for continuous variables and Pearson’s chi-squared test for categorical variables. Median overall survival (mOS) and median progression free survival (mPFS) were estimated from Kaplan-Meier curves. Unadjusted and adjusted Cox proportional hazard models were used to compute hazard ratios (HR) to assess the effect of exposures on mOS. Tumor reduction was determined from cross-sectional imaging reports. RECIST 1.1 calculations were done on the available radiographic images.

This study was approved by the University of Pennsylvania Health System Institutional Review Board.

## 3 Results

### 3.1 Baseline characteristics

47 patients met criteria for inclusion; baseline demographics and treatment characteristics are reported in Table 2. Overall, the median age was 61.2 years; the cohort was 46.8% female and 76.6% white. Approximately half had smoking exposure (44.7%) or self-reported known or suspected asbestos exposure (55.3%), and the majority (76.6%) had epithelioid histology, consistent with previously reported data.

**Table 2:** Patient demographics.

*Variable*		*Overall*	*Neoadjuvant*	*Adjuvant*	*Surgery Only*	*No Surgery*
		*N* = 47	*n* = 12	*n* = 8	*n* = 7	*n* = 20
**Age at diagnosis**	median, in years	61.2	59.5	58.1	62.3	63.2
**Sex**	% female	46.8%	50.0%	62.5%	42.9%	40.0%
**Race**	% white	76.6%	83.3%	87.5%	57.1%	75.0%
	% black	6.4%	0%	12.5%	14.3%	5.0%
	% other race	17.0%	16.7%	0%	28.6%	20.0%
**Smoking**	% never smoker	55.3%	83.3%	37.5%	71.4%	40.0%
**Asbestos**	% with exposure	55.3%	58.3%	37.5%	42.9%	65.0%
**ECOG PS**	0	15 (40.5%)	6 (60.0%)	3 (75.0%)	3 (50.0%)	3 (17.6%)
	1	17 (45.9%)	4 (40.0%)	1 (25.0%)	2 (33.3%)	10 (58.8%)
	2+	5 (13.5%)	0 (0%)	0 (0%)	1 (16.7%)	4 (23.5%)
**Histology**	Epithelioid	36 (76.6%)	11 (91.7%)	7 (87.5%)	6 (85.7%)	12 (60.0%)
	Biphasic	2 (4.3%)	0 (0%)	0 (0%)	1 (14.3%)	1 (5.0%)
	Sarcomatoid	5 (10.6%)	1 (8.3%)	1 (12.5%)	0 (0%)	3 (15.0%)
	Other/Unknown	4 (8.5%)	0 (0%)	0 (0%)	0 (0%)	3 (20.0%)
**Surgical debulking**		25 (53.2%)	10 (83.3%)**	8 (100%)	7 (100%)	0 (0%)
**HIPEC** ^#^		19 (76.0%)	9 (90.0%)	5 (62.5%)	5 (71.4%)	0 (0%)
**Systemic chemotherapy** ^^^		37 (78.7%)	12 (100%)	8 (100%)	2 (28.6%)	15 (75.0%)
	Adjuvant	8 (17.0%)				
	Neoadjuvant	12 (25.6%)				
	Palliative	24 (51.1%)	3 (25.0%)	4 (50.0%)	2 (28.6%)	15 (75.0%)

Due to rounding, percentages may not add up to 100%; percentages are in relation to known data points.

ECOG PS = Eastern Cooperative Oncology Group Performance Status. HIPEC = hyperthermic intraperitoneal chemotherapy.

**Of patients who received neoadjuvant systemic therapy, 2 patients were lost to follow-up. These patients are NOT included in the “No Surgery” group.

^#^HIPEC is calculated as percentage of patients who received surgery.

^^^This represents systemic chemotherapy received in any context; patients may receive more than one modality. For example, a patient who received surgery and then develops recurrent disease may receive both neoadjuvant and palliative chemotherapy.

Approximately half (*n* = 25/47, 53.2%, with 2 surgical candidates lost to follow-up) had undergone cytoreductive surgery at time of analysis; most of these patients (19/25, 76.0%) also underwent HIPEC, with the remainder deferring HIPEC for heterogeneous reasons (e.g., initial debulking surgery for presumed gynecologic malignancy prior to DMPM diagnosis, tumor burden limiting HIPEC). Among patients who were planned for surgery, 12 received neoadjuvant chemotherapy, 8 received adjuvant chemotherapy, and 7 underwent surgery alone without systemic chemotherapy– 1 patient received both neoadjuvant and adjuvant therapy and is only included in the neoadjuvant group as listed above, as well as for all analyses. Non-surgical patients had either disease that was not amenable to surgical resection or comorbidities that limited candidacy for surgery; these patients were offered first-line palliative chemotherapy, when appropriate.

### 3.2 Baseline characteristics: Neoadjuvant vs adjuvant chemotherapy groups

Neoadjuvant and adjuvant group patient pre-treatment characteristics were similar (Table 3), except for smoking status, where patients who received neoadjuvant chemotherapy were more likely to be never smokers. All patients had a pre-treatment ECOG PS of 0–1, and the majority had a pre-treatment CCI index of 0–1. Pre-treatment labs were similar, including platelets, platelet-to-lymphocyte ratio, neutrophil-to-lymphocyte ratio, and serum albumin, all of which have been reported as values associated with survival in DMPM [19–21]. Finally, disease burden, as measured by largest peritoneal implant size and presence or absence of ascites, were similar between groups.

**Table 3:** Comparison of neoadjuvant and adjuvant patients.

*Variable*		*Neoadjuvant*	*Adjuvant*	
		*n* = 12	*n* = 8	
**Age at diagnosis**	median, in years	59.5	58.1	*p* = 0.80
**Sex**	% female	50.0%	62.5%	*p* = 0.58
**Race**	% white	83.3%	87.5%	*p* = 0.24
	% black	0%	12.5%	
	% other race	16.7%	0%	
**Smoking**	% never smoker	83.3%	37.5%	*p* = 0.04
**Asbestos**	% with exposure	58.3%	37.5%	*p* = 0.36
**ECOG PS**	0	6 (60.0%)	3 (75.0%)	*p* = 0.32
	1	4 (40.0%)	1 (25.0%)	
	2+	0 (0%)	0 (0%)	
**CCI prior to Diagnosis**	0–1	7 (58.3%)	4 (66.7%)	*p* = 0.66
	2–3	4 (33.3%)	2 (33.3%)	
	4+	1 (8.3%)	0 (0%)	
**Pre-treatment labs (mean)**	platelets (x1000/μL)	321.0 (*n* = 10)	317.7 (*n* = 3)	*p* = 0.96
	ANC (x1000/μL)	5.31 (*n* = 10)	5.65 (*n* = 2)	*p* = 0.87
	ALC (x1000/μL)	1.29 (*n* = 9)	1.45 (*n* = 2)	*p* = 0.63
	platelets/ALC	273.1 (*n* = 9)	243.8 (*n* = 2)	*p* = 0.65
	ANC/ALC	4.63 (*n* = 9)	4.21 (*n* = 2)	*p* = 0.86
	albumin (g/dL)	4.08 (*n* = 8)	3.85 (*n* = 2)	*p* = 0.18
**Largest peritoneal implant**	<2.5cm or diffuse	9 (75.0%)	4 (67.0%)	*p* = 0.6
	2.5cm to 5cm	1 (8.3%)	0 (0%)	
	>5cm	2 (16.7%)	2 (33.0%)	
**Pre-treatment ascites**	None	2 (16.7%)	3 (50.0%)	*p* = 0.12
	Present	10 (83.3%)	3^a^ (50.0%)	

^a^Pre-treatment images were not available for 2 patients. ECOG PS = Eastern Cooperative Oncology Group Performance Status.

CCI = Charlson Comorbidity Index. ANC = absolute neutrophil count. ALC = absolute lymphocyte count. Due to rounding, percentages may not add up to 100%; percentages are in relation to known data points. *p* values represent comparison between groups, using 2-tailed heteroscedastic Student’s t-test for continuous variables and Pearson’s chi-squared test for categorical variables.

### 3.3 Systemic therapy

Most patients (37/47, 78.7%) received systemic chemotherapy, including the perioperative setting (*n* = 20), first-line palliative setting (*n* = 15), and recurrent setting after initial curative-intent surgery (*n* = 2). In the neoadjuvant group, median cycles given was four (range 1–7 cycles), with all patients receiving platinum agent (cisplatin or carboplatin) and pemetrexed (one patient received cetuximab in addition). In the adjuvant group, median cycles given was five (range 4–8, not including one patient receiving prolonged course of maintenance pemetrexed), with 7/8 patients receiving a platinum agent and pemetrexed, and 1/8 patients receiving pemetrexed alone. All adjuvant chemotherapy was initiated within 3 months of surgery (median 35 days, range 25–92 days).

In general, systemic therapy was well tolerated in the neoadjuvant group, with most patients experiencing expected chemotherapy-related toxicity, such as fatigue and gastrointestinal symptoms (anorexia, nausea, diarrhea). Two patients were lost to follow-up, and of the remaining 10 patients, two had complications during their course: one was switched from cisplatin to carboplatin for severe nausea (and otherwise completed therapy on time), and another had a delay in surgery due to post-operative functional limitations after an interim staging laparoscopy; it was unclear whether this was causally related to the neoadjuvant chemotherapy versus the laparoscopic procedure. In the adjuvant group, 4/8 patients had complications with their chemotherapy course: two patients were switched from cisplatin to carboplatin for toxicities (both completed courses on time), one stopped due to worsening renal function (renal function improved off of chemotherapy), and one stopped due to a post-operative small bowel obstruction. There were no further delays in therapy, dose reductions in chemotherapy, delays in surgery, or unplanned hospital admissions in either group.

**Fig 1:**
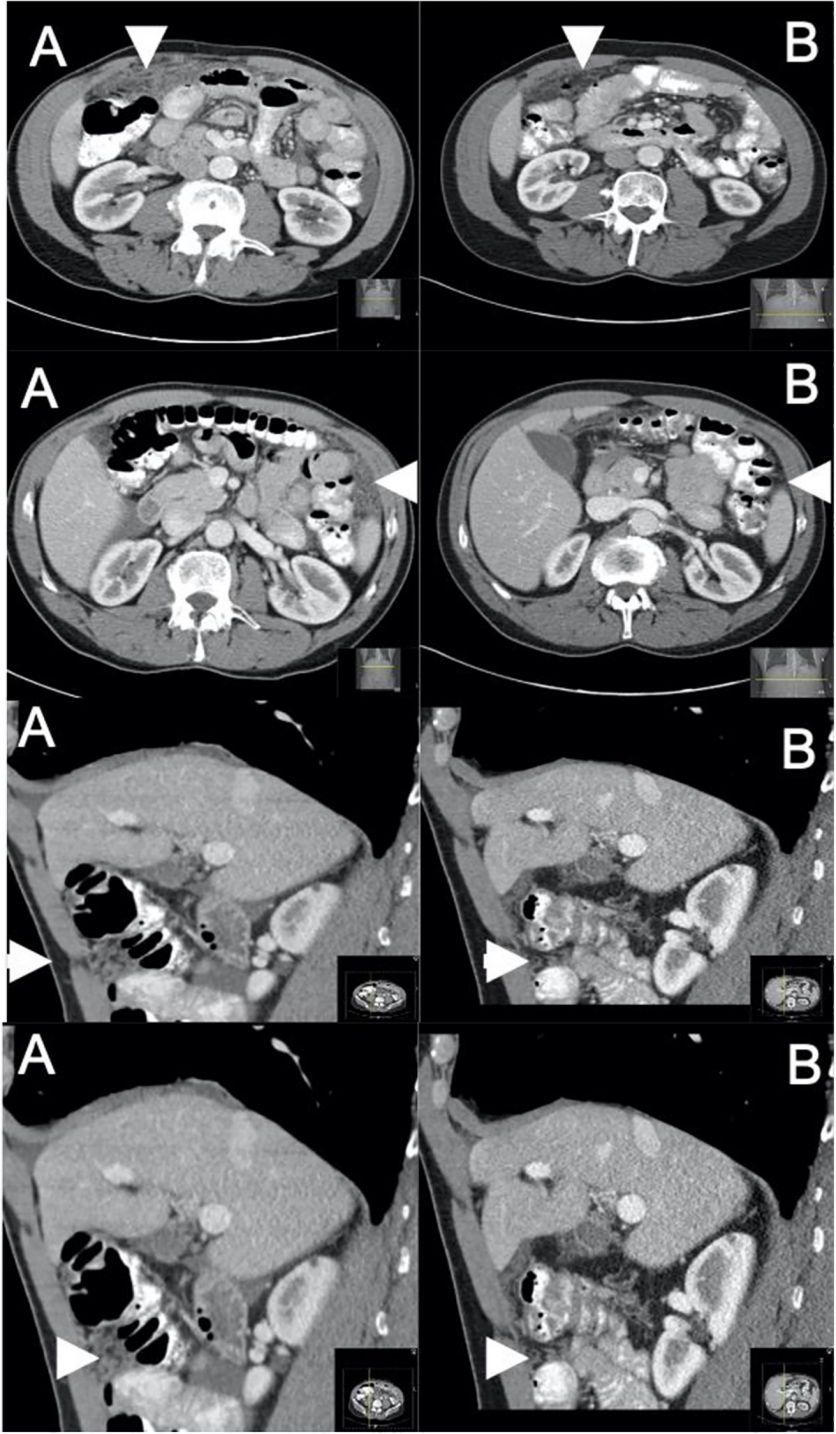
Partial response to neoadjuvant chemotherapy. Patient with DMPM with initial CT images prior to administration of systemic chemotherapy (A) and after six cycles of neoadjuvant carboplatin and pemetrexed (B), with marked partial response to therapy.

Among the 10 neoadjuvant patients who successfully followed up through surgery, CT scans revealed mostly stable to improved disease during the pre-operative period. Images from baseline and at least 1 follow up scan were only available for 4 patients (1 complete response, 2 partial responses, 1 with complete response in non-target lesions given no measurable disease for target lesions by RECIST v1.1); the remaining patients only had imaging reports available, which were reviewed for interval improvement, stability, or progression of disease. Using these approaches, only 1/10 patients experienced disease progression during this neoadjuvant period. Cross sectional imaging from a representative patient receiving neoadjuvant chemotherapy is provided in Fig 1.

### 3.4 Surgical course: Neoadjuvant vs adjuvant chemotherapy groups

Within the neoadjuvant group, 10 (83.3%) successfully proceeded to their planned debulking surgery; 2 (16.7%) were lost to follow-up. Median length of stay for the index hospitalization for surgery was 8.5 days (range 5–12 days), and all operative times were between 3.5 and 6 hours (median 5 hours, 16 minutes). These were roughly equivalent to the adjuvant surgical group, though data for that group was more sparse (median LOS 7 days, median surgical time 4 hours, 24 minutes, both *p*s > 0.05 by two-tailed Student’s t-test).

In addition, 9/10 (90.0%) neoadjuvant patients underwent HIPEC with their surgeries, a higher proportion compared to 5/8 (62.5%) adjuvant patients and 5/7 (71.4%) surgery-only patients. No significant toxicities or adverse events were noted in the post-operative setting with neoadjuvant patients, with fatigue, weight loss, and bloating being the most common symptoms reported after surgery. Among adjuvant patients, one patient developed a post-operative empyema, while another developed a bowel obstruction within a few months of surgery.

### 3.5 Overall survival

**Fig 2:**
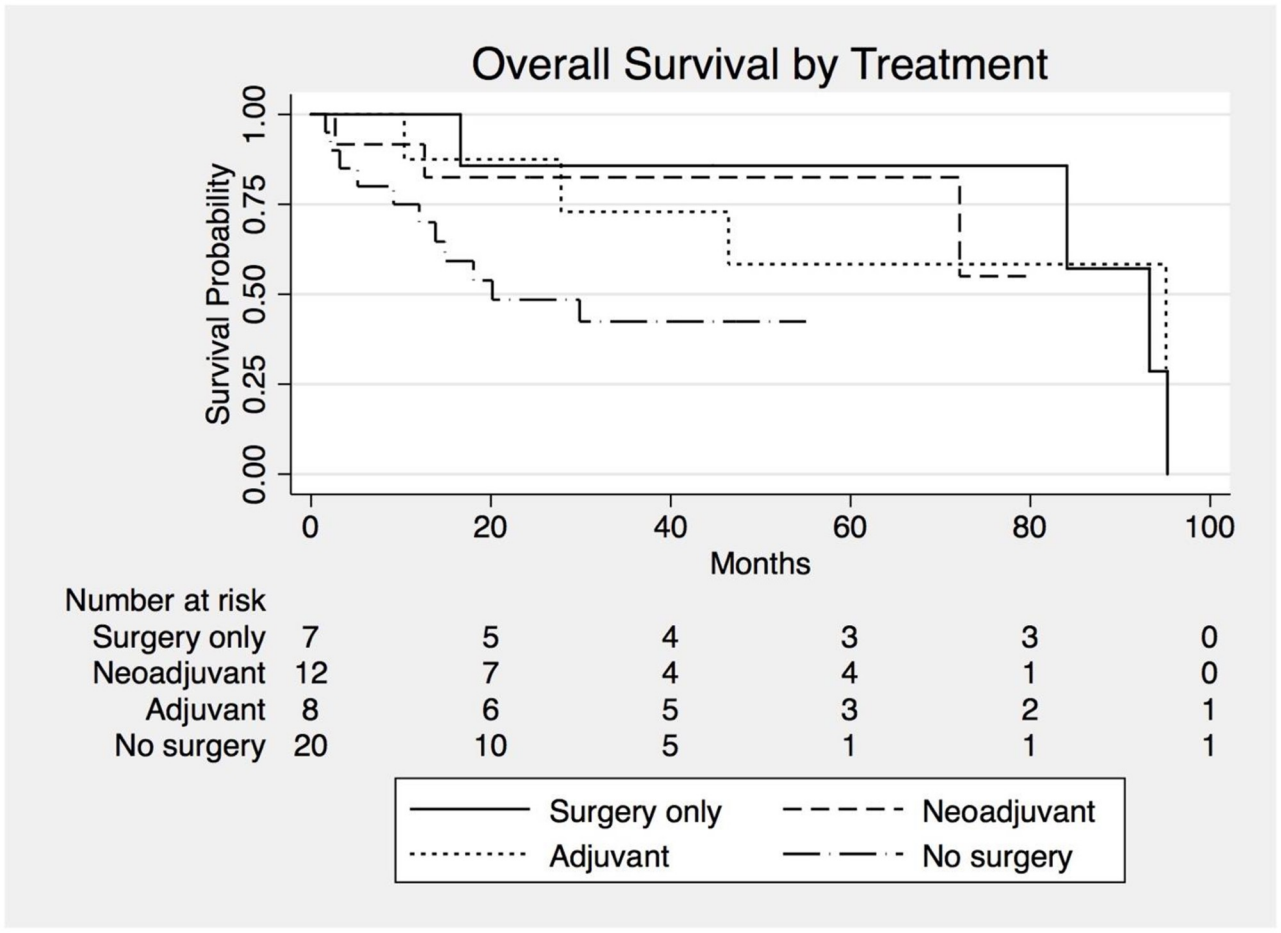
Overall survival by type of therapy. Kaplan-Meier curves for overall survival by surgery and perioperative chemotherapy.

Survival data are reported in Table 4. Median overall survival (mOS) for all participants was 84.1 months. Among surgical patients, HIPEC was not associated with a statistically significant difference in outcomes (HR 0.48, 95% CI 0.13–1.69, *p* = 0.25). No statistically significant difference was seen in mOS between treatment groups (Table 4, Fig 2). There was no difference in mOS between neoadjuvant and adjuvant chemotherapy groups among patients who received surgery (mOS not reached vs 95.1 months (HR 0.89, 95% CI 0.18–4.5, *p* = 0.89). When including smoking status, which is not balanced between the two groups, in a multivariable model, there is still no statistical advantage for neoadjuvant compared to adjuvant chemotherapy (HR 0.69, 95% CI 0.09–5.4, *p* = 0.729). Finally, when analysis is restricted only to patients with epithelioid histology, these findings persist (mOS NR vs 95.1 months, HR 1.01, 95% CI 0.14–7.3, *p* = 0.99).

**Table 4:** Median overall survival (mOS), by variable.

*Variable*		*n*	*mOS in months*	
**Overall**		47	84.1 (95% CI 27.8–95.1)	
**Treatment Group**				
	Neoadjuvant	12	NR (95% CI 12.6 –NA)	*p* = 0.30
	Adjuvant	8	95.1 (95% CI 10.4 –NA)	
	Surgery Only	7	93.2 (95% CI 16.6 –NA)	
	No Surgery	20	20.2 (95% CI 9.2 –NA)	
**Surgical Debulking**				
	No	22	20.2 (95% CI 9.18 –NA)	*p* = 0.03
	Yes	25	93.2 (95% CI 46.4–95.2)	
**HIPEC** ^a^				
	No	7	46.4 (95% CI 12.6 –NA)	*p* = 0.25
	Yes	18	95.1 (95% CI 72.1 –NA)	

^a^Among patients who underwent surgery.

HIPEC = hyperthermic intraperitoneal chemotherapy. 95% CI = 95% confidence interval. NA = not achieved. *p* values derived from log rank test.

## 4 Discussion

The role and timing of chemotherapy in surgically resectable DMPM is still an active area of research, with no consensus “standard of care.” Previous studies have shown similar or decreased overall survival for neoadjuvant compared to adjuvant chemotherapy. However, patient selection may have biased the results in heterogenous settings including multiple institutions. Patients selected for a neoadjuvant chemotherapy approach in these studies may have been suboptimal surgical candidates leading to the choice of neoadjuvant chemotherapy to improve surgical candidacy but also confounding overall survival. Our study presents a single institution’s experience with a preferentially neoadjuvant approach, therefore potentially decreasing this bias.

Our study demonstrates that neoadjuvant chemotherapy is a safe and viable treatment option for patients with DMPM. Consistent with previous reports, we found that patients undergoing cytoreductive surgery (CRS) have better clinical outcomes compared to those receiving chemotherapy alone. The timing and benefit of perioperative chemotherapy is less impactful, and we found no difference in mOS for surgical candidates who received neoadjuvant versus adjuvant chemotherapy. On the contrary, we observed that neoadjuvant chemotherapy may offer benefit for surgical and HIPEC candidacy, offering some rationale for its choice over adjuvant chemotherapy.

Although not statistically significant, a higher proportion of patients in the neoadjuvant group were able to receive CRS with HIPEC compared to the adjuvant group, possibly due to superior debulking after neoadjuvant chemotherapy. HIPEC is generally only considered when surgical debulking down to 1–3 mm residual tumor thickness is possible given its limited efficacy in tumors with greater thickness [22]; patients receiving adjuvant chemotherapy without the preoperative effects of tumor shrinkage may derive less benefit from HIPEC itself. Finally, adjuvant chemotherapy can be delayed due to surgical complications, causing further interruptions in therapy. The adjuvant group had a higher rate of issues with chemotherapy (50% vs 20% neoadjuvant), with one patient overtly stopping treatment due to post-surgical small bowel obstruction. We did not observe any unexpected adverse events or toxicity following surgery in the neoadjuvant group.

Thus, if perioperative chemotherapy is to be considered, our center continues to advocate for its use in the neoadjuvant setting, given our institutional experience of its tolerability, as well as its potential for improved surgical resectability, candidacy for HIPEC, and symptom burden. Our study is limited by its retrospective nature and small sample size, and the study reports exclusively on patients evaluated at our institution, a tertiary care referral center for DMPM, and therefore may lack external validity across a wider population and in the community where there is often less comfort with the surgical interventions used. In addition, there may have been factors that led some patients to undergo neoadjuvant therapy, while others proceeded straight to surgery. We attempted to expose these factors through comparison of baseline and treatment characteristics, but only prospective randomization can truly account for unmeasured factors. Nevertheless, based on the factors measured, we showed that the baseline characteristics were similar between adjuvant and neoadjuvant groups including age, performance status, and largest peritoneal implant, which are all factors that may influence surgical candidacy (Table 2). The challenge of studying rare cancers have been well-documented, both in general [23, 24] and in reference to malignant mesothelioma specifically [25], highlighting the need for multicenter and multinational collaborative efforts. Given the difficulty in developing standardized treatments for DMPM, we believe that the outcomes of our cohort, despite its small, retrospective nature, offer value to the field and its patients. Further prospective studies are necessary, and we aim to engage with and collaborate with colleagues at other institutions to do so.

In summary, we found that neoadjuvant chemotherapy was not associated with inferior overall survival compared to adjuvant chemotherapy in DMPM patients with similar baseline characteristics. In addition, we showed that neoadjuvant chemotherapy did not compromise surgical candidacy and may have increased the proportion of patients eligible for effective HIPEC therapy. Future prospective studies should focus on clarifying the role and timing of perioperative chemotherapy. Furthermore, as further advances in the treatment of DMPM are made–such as the use of immune checkpoint inhibitors [26] and the identification of potentially targetable mutations [27]–it will be important to define their utility in the perioperative setting.

## Supporting information

S1 File (XLSX)
