# Correction: A single-center retrospective cohort study of perioperative systemic chemotherapy in diffuse malignant peritoneal mesothelioma

**DOI:** 10.1371/journal.pone.0313663

**Published:** 2024-11-07

**Authors:** The PLOS ONE Editors

## Notice of Republication

This article [1] was republished on October 30, 2024, to address an issue identified post-publication and to provide an updated version of S1 File. Please download this article again to view the correct version.
